# Alcohol Consumption During Pregnancy Among Women Aged 18–49 Years — United States, 2021–2024

**DOI:** 10.15585/mmwr.mm7522a2

**Published:** 2026-06-11

**Authors:** Shawn A. Thomas, Lucas K. Gosdin, Mishka Terplan, Shin Y. Kim, Nicholas P. Deputy

**Affiliations:** ^1^Division of Birth Defects and Infant Disorders, National Center on Birth Defects and Developmental Disabilities, CDC; ^2^Epidemic Intelligence Service, CDC; ^3^Friends Research Institute, Baltimore, Maryland.

**Summary**

**What is already known about this topic?**

Alcohol consumption during pregnancy can increase the risk for adverse pregnancy and birth outcomes. No amount of alcohol consumption during pregnancy is known to be safe.

**What is added by this report?**

According to 2021–2024 data from the Behavioral Risk Factor Surveillance System, 15.2% of pregnant women in the United States reported current drinking, 4.9% reported binge drinking, and 2.2% reported heavy drinking during the past 30 days.

**What are the implications for public health practice?**

Clinical approaches, such as routine screening for alcohol consumption and mental health conditions during pregnancy, and community-level approaches, such as point-of-sale warning signs or alcohol sales taxes, might help reduce alcohol consumption during pregnancy and its associated adverse pregnancy and birth outcomes.

## Abstract

Alcohol consumption during pregnancy can cause adverse birth outcomes and fetal alcohol spectrum disorders. One U.S. study identified a slight increasing trend in alcohol consumption during pregnancy from 2011 to 2018. During 2018–2020, 13.5% of pregnant women reported current drinking; more recent estimates are unavailable. CDC analyzed 2021–2024 Behavioral Risk Factor Surveillance System data to estimate prevalence of self-reported current drinking (one or more alcoholic drinks during the past 30 days), binge drinking (four or more alcoholic drinks on at least one occasion during the past 30 days), and heavy drinking (eight or more alcoholic drinks within 1 week during the past 30 days) among U.S. pregnant women aged 18–49 years. Multivariable regression was used to estimate adjusted prevalence ratios and identify correlates of alcohol consumption during pregnancy. Among U.S. pregnant women, 15.2% reported current drinking, 4.9% reported binge drinking, and 2.2% reported heavy drinking during the past 30 days. Higher prevalences of alcohol consumption were observed among pregnant women who were not married and those with frequent mental distress. Alcohol consumption during pregnancy remains a public health concern. Both clinical and community interventions might help reduce alcohol consumption during pregnancy and its associated adverse health outcomes.

## Introduction

Alcohol consumption during pregnancy can cause adverse pregnancy and birth outcomes, including miscarriage and stillbirth, and fetal alcohol spectrum disorders, a group of lifelong behavioral, intellectual, and physical conditions. No amount of alcohol consumption during pregnancy is known to be safe; higher frequency and intensity of alcohol consumption during pregnancy are linked with higher risk for adverse pregnancy and birth outcomes (*1*). One previous study found a slight increasing trend in the prevalence of current drinking and binge drinking during pregnancy from 2011 to 2018, although increases were not consistently observed throughout that period (*2*). A recent study found current drinking and binge drinking prevalences of 13.5% and 5.2%, respectively, during 2018–2020 (*3*); more recent prevalence estimates are not available. To provide more recent estimates of alcohol consumption among pregnant women, CDC analyzed Behavioral Risk Factor Surveillance System (BRFSS) data to assess prevalences and correlates of self-reported current drinking, binge drinking, and heavy drinking among pregnant women aged 18–49 years in the United States during 2021–2024.

## Methods

### Data Source 

BRFSS is an annual, state-based, random-digit–dialed telephone survey of health-related behaviors among noninstitutionalized adults aged ≥18 years in the United States and participating territories. To generate reliable prevalence estimates for this analysis, 2021–2024 BRFSS data from 50 U.S. states* and the District of Columbia were pooled. Across jurisdictions, median BRFSS response rates were 44.0% (range = 23.5%–60.5%) in 2021, 45.0% (22.8%–66.8%) in 2022, 44.7% (21.7%–63.1%) in 2023, and 43.9% (30.7%–64.8%) in 2024.^†^

### Survey Measures 

Respondents who reported that their sex at birth was female were asked whether they were currently pregnant; information on trimester of pregnancy was not collected. Current drinking (one or more alcoholic drinks during the past 30 days) and binge drinking (four or more alcoholic drinks on at least one occasion during the past 30 days)^§^ were defined based on the 2020–2025 Dietary Guidelines for Americans; heavy drinking (eight or more alcoholic drinks in a 1-week period during the past 30 days)^¶^ was classified based on CDC definitions. Sociodemographic and health characteristics examined included age, race and ethnicity, education, employment status, marital status, having a usual health care provider,** and experiencing frequent mental distress.^††^

### Statistical Analysis

Among pregnant women aged 18–49 years with complete data for current drinking (8,579, unweighted), binge drinking (8,546, unweighted), or heavy drinking (8,550, unweighted) prevalences and 95% CIs were estimated overall, by survey year, and by sociodemographic and health characteristics. Because of small sample sizes for binge drinking and heavy drinking, only current drinking could be examined by geographic region. Rao-Scott chi-square tests were used to identify differences by survey year and geographic region. p-values <0.05 were considered statistically significant. Multivariable modified Poisson regression models with log link and robust SEs were used to estimate adjusted prevalence ratios (aPRs) and 95% CIs for associations between sociodemographic and health characteristics and current drinking, binge drinking, and heavy drinking. Estimates were considered statistically significant when 95% CIs excluded 1.0 or were nonoverlapping. Respondents with missing values for sociodemographic or health characteristics were excluded from the respective analyses.^§§^ Data were weighted to state-level population estimates and pooled to represent regional and national estimates. Analyses used SAS statistical software (version 9.4; SAS Institute); sample weights and design variables were incorporated and adjusted as recommended by BRFSS to account for the complex survey design and pooling of multiple years. This activity was reviewed by CDC, deemed not research, and conducted consistent with applicable federal law and CDC policy.^¶¶^

## Results

### Prevalence of Alcohol Consumption Among Pregnant Women

Among U.S. pregnant women aged 18–49 years during 2021–2024, 15.2% reported current drinking, 4.9% reported binge drinking, and 2.2% reported heavy drinking during the past 30 days (Table). Among those reporting current drinking, 33.2% also reported binge drinking and 14.7% also reported heavy drinking. Prevalences did not differ significantly by survey year (Table).

**TABLE:** Estimated prevalence* and adjusted prevalence ratios of current drinking, binge drinking, and heavy drinking reported by pregnant women aged 18–49 years, by selected characteristics — Behavioral Risk Factor Surveillance System, United States, 2021−2024^†^

Characteristic	Current drinking^§^		Binge drinking^¶^		Heavy drinking**	
	% (95% CI)	aPR^††^ (95% CI)	% (95% CI)	aPR^††^ (95% CI)	% (95% CI)	aPR^††^ (95% CI)
**Total**	**15.2 (13.6–16.8)**	**—**	**4.9 (4.0–5.8)**	**—**	**2.2 (1.6–2.7)**	**—**
**Survey year**						
2021	15.8 (12.6–19.1)	—	5.6 (3.0–8.2)**^§§^**	—	2.1 (1.0–3.1)**^§§^**	—
2022	15.4 (12.6–18.1)	—	5.0 (3.5–6.5)	—	2.6 (1.5–3.6)**^§§^**	—
2023	15.1 (12.0–18.3)	—	5.5 (3.7–7.3)	—	NA^¶¶^	—
2024	14.5 (11.1–17.8)	—	3.5 (2.3–4.8)	—	1.2 (0.7–1.8)**^§§^**	—
**Age group, yrs**						
18–24	18.0 (14.3–21.7)	0.9 (0.7–1.1)	7.7 (4.9–10.4)	1.2 (0.7–2.0)	3.1 (1.7–4.4)**^§§^**	0.8 (0.4–1.5)
25–29	12.0 (9.7–14.2)	0.6 (0.5–0.8)	3.7 (2.4–4.9)	0.6 (0.4–1.1)	1.5 (0.6–2.3)^§§^	0.5 (0.2–1.1)
30–34	10.5 (8.3–12.8)	0.5 (0.4–0.7)	2.3 (1.6–3.1)	0.4 (0.2–0.6)	NA^¶¶^	NA^¶¶^
35–49	21.4 (17.6–25.2)	Ref	6.1 (4.3–8.0)	Ref	2.8 (1.6–4.0)^§§^	Ref
Race and ethnicity***						
Black or African American	17.9 (13.0–22.8)	1.1 (0.7–1.6)	7.6 (4.8–10.5)	1.4 (0.8–2.5)	3.7 (1.7–5.6)**^§§^**	1.9 (0.9–4.0)
White	15.0 (13.2–16.9)	1.1 (0.8–1.5)	4.1 (3.3–5.0)	1.0 (0.6–1.9)	2.1 (1.2–2.9)**^§§^**	1.6 (0.8–3.2)
Hispanic or Latino	12.5 (9.4–15.7)	Ref	4.2 (2.5–6.0)**^††^**	Ref	1.5 (0.8–2.2)**^§§^**	Ref
Other	19.9 (14.2–25.7)	1.4 (0.9–2.1)	NA^¶¶^	NA^¶¶^	NA^¶¶^	NA^¶¶^
**Education**						
High school diploma or less	12.9 (10.3–15.5)	Ref	4.9 (3.1–6.6)	Ref	1.8 (1.1–2.6)**^§§^**	Ref
Some college	15.8 (12.7–19.0)	1.2 (0.9–1.6)	5.1 (3.7–6.5)	1.1 (0.7–1.8)	3.0 (1.6–4.4)**^§§^**	1.6 (0.8–3.0)
College degree	18.0 (15.7–20.3)	1.5 (1.2–2.0)	4.8 (3.5–6.1)	1.4 (0.8–2.3)	1.8 (1.0–2.6)^§§^	1.2 (0.5–2.5)
**Employment status**						
Employed	17.1 (15.1–19.1)	1.2 (1.0–1.6)	5.0 (3.9–6.1)	1.1 (0.7–1.7)	2.2 (1.5–3.0)	1.1 (0.6–1.9)
Not employed	12.8 (10.3–15.3)	Ref	4.8 (3.2–6.4)	Ref	2.2 (1.3–3.0)**^§§^**	Ref
**Marital status**						
Married	12.0 (10.1–13.9)	Ref	3.1 (2.1–4.2)	Ref	1.3 (0.6–2.0)**^§§^**	Ref
Not married	19.1 (16.5–21.7)	1.8 (1.4–2.2)	7.0 (5.4–8.6)	2.2 (1.3–3.7)	3.1 (2.2–4.0)	2.0 (1.1–3.6)
**Has a usual health care provider**						
Yes	16.0 (14.2–17.9)	Ref	5.0 (3.9–6.1)	Ref	2.2 (1.5–2.8)	Ref
No	12.2 (9.5–15.0)	0.8 (0.6–1.0)	4.2 (2.6–5.8)	0.7 (0.4–1.3)	2.1 (0.9–3.3)**^§§^**	1.0 (0.5–2.2)
**Frequent mental distress** ^†††^						
Yes	26.0 (21.3–30.8)	1.8 (1.4–2.3)	9.0 (6.6–11.4)	1.8 (1.2–2.6)	5.7 (3.7–7.7)	3.0 (1.7–5.0)
No	13.3 (11.7–15.0)	Ref	4.1 (3.1–5.2)	Ref	1.6 (1.0–2.2)	Ref

**Abbreviations**: aPR = adjusted prevalence ratio; NA = not available; Ref = referent group.

* Percentages are weighted to represent national estimates of the noninstitutionalized U.S. adult population.

^†^ Florida (2021), Kentucky (2023), Pennsylvania (2023), and Tennessee (2024) did not collect sufficient data to meet minimum requirements for inclusion in the Behavioral Risk Factor Surveillance System public use data set for those years.

^§^ Defined as self-reported consumption of one or more alcoholic drinks during the past 30 days.

^¶^ Defined as self-reported consumption of four or more alcoholic drinks on at least one occasion during the past 30 days.

** Defined as self-reported consumption of eight or more alcoholic drinks in 1 week during the past 30 days.

^††^ aPRs generated from complete-case multivariable modified Poisson regression models are adjusted for age, race and ethnicity, education, employment status, marital status, usual health care provider, and frequent mental distress.

^§§^ Prevalence estimate might be unstable because the relative SE is 0.2–0.3.

^¶¶^ Prevalence estimate or corresponding prevalence ratio is suppressed because the relative SE for the prevalence estimate is >0.3.

*** Persons of Hispanic or Latino (Hispanic) origin might be of any race but are categorized as Hispanic; all racial groups are non-Hispanic.

^†††^ Defined as reporting ≥14 days of poor mental health during the past 30 days.

### Characteristics Associated with Alcohol Consumption Among Pregnant Women

Several characteristics were statistically significantly associated with current drinking, binge drinking, or heavy drinking (Table). For example, pregnant women who were not married had approximately twice the prevalence of current drinking (aPR = 1.8), binge drinking (aPR = 2.2), and heavy drinking (aPR = 2.0) compared with those who were married. In addition, pregnant women reporting frequent mental distress had approximately twice the prevalence of current drinking (aPR = 1.8) and binge drinking (aPR = 1.8) and three times the prevalence of heavy drinking (aPR = 3.0) compared with those who did not report frequent mental distress. Significant differences existed by geographic region for current drinking (p = 0.04): pregnant women living in U.S. Department of Health and Human Services (HHS) Region 1 (19.9%) had a higher prevalence of current drinking than did those living in HHS Region 6 (10.4%), HHS Region 7 (11.8%), and HHS Region 8 (12.4%) (Figure).

**FIGURE:**
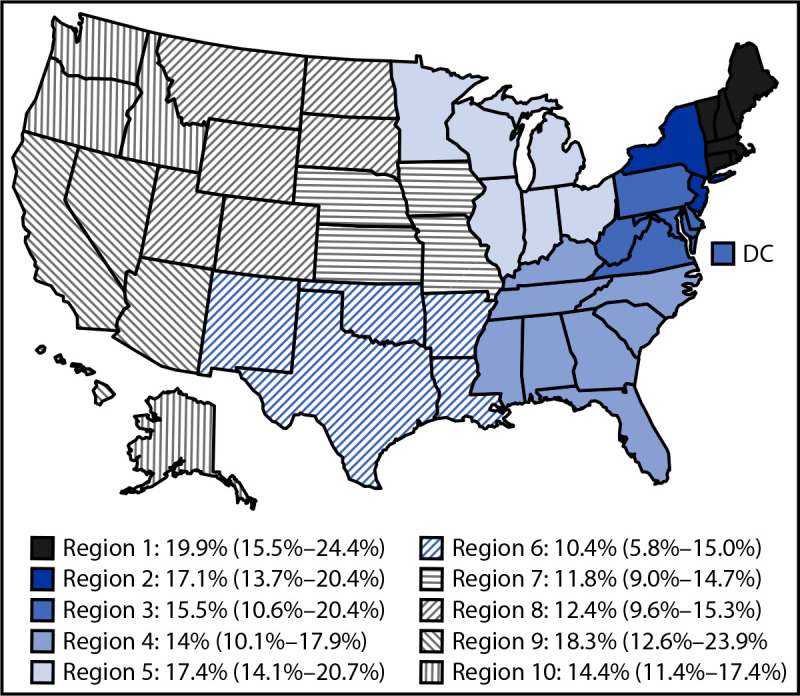
Estimated prevalence* of current drinking^†^ during the past 30 days reported by pregnant women aged 18–49 years (N = 8,579), by U.S. Department of Health and Human Services region^§^ — Behavioral Risk Factor Surveillance System, United States, 2021–2024 **Abbreviation:** DC = District of Columbia. * Percentages (with 95% CIs) are weighted to represent national estimates of the noninstitutionalized U.S. adult population. Estimate for region 6 might be unstable because the relative SE is 0.2–0.3. Florida (2021), Kentucky (2023), Pennsylvania (2023), and Tennessee (2024) did not collect sufficient data to meet minimum requirements for inclusion in the Behavioral Risk Factor Surveillance System public use data set for those years. ^†^ Defined as self-reported consumption of one or more alcoholic drinks during the past 30 days. ^§^ U.S. Department of Health and Human Services regions

## Discussion

Alcohol consumption during pregnancy remains a public health concern in the United States. The estimated prevalence of current drinking among pregnant women during 2021–2024 (15.2%) appears to be higher than it was during 2018–2020 (13.5%) (*3*), suggesting an ongoing need for clinical and community-level interventions.

Regional differences in current drinking during pregnancy observed in this analysis are generally consistent with patterns of current drinking observed among the general U.S. population. Geographic differences highlight the importance of tailoring public health interventions to account for local context and cultural norms when addressing alcohol consumption during pregnancy. Community-level approaches that focus on pregnant women, such as point-of-sale signs with information about adverse outcomes known to be associated with alcohol consumption during pregnancy (*4*) or that focus on the general population, such as sales taxes on alcoholic beverages (*5*), might help reduce prenatal alcohol exposure and its associated adverse health outcomes.

Pregnant women who were not married or who reported experiencing frequent mental distress had approximately two to three times the prevalence of current drinking, binge drinking, and heavy drinking compared with those who were married or did not experience frequent distress, consistent with findings from a previous study of current drinking and binge drinking during pregnancy (*3*). Studies suggest alcohol consumption might be used as a coping method to relieve stress and manage negative feelings, although alcohol consumption might alter or exacerbate stress pathways (*6*). Marital and other cohabitation statuses have been associated with reduced substance use, which might be explained in part by social support, although relationship quality and other factors might influence this association (*7*). These findings reinforce the importance of integrating behavioral health screening, treatment, and other support into prenatal care. The U.S. Preventive Services Task Force in 2018 recommended screening adults for unhealthy alcohol use, including alcohol use during pregnancy, and brief counseling to address unhealthy use (*8*). In addition, the American College of Obstetricians and Gynecologists recommends screening all pregnant women for anxiety and depression during prenatal care visits, emphasizing the importance of having systems in place, when needed, to ensure access to appropriate services (*9*).

### Limitations

The findings in this report are subject to at least five limitations. First, some survey respondents who became pregnant within 30 days of survey administration might have reported alcohol use that occurred before, not during, pregnancy; this might have resulted in overestimates of alcohol consumption during pregnancy because studies conducted among women who delivered live infants have found alcohol consumption typically decreases when women realize they are pregnant (*10*). Second, self-reported alcohol consumption might be subject to misclassification related to social desirability and recall biases, which might have resulted in underestimates of consumption; in addition, because BRFSS questions do not capture all alcohol consumption patterns, heavy drinking results might be underestimates. Third, self-reported pregnancy status might be misclassified because early pregnancies might be unrecognized and unreported. Fourth, temporality is unknown for observed associations between health characteristics and alcohol consumption because data are cross-sectional. Finally, this analysis did not include an evaluation of patterns of alcohol consumption throughout pregnancy because information on trimester of pregnancy was not collected.

### Implications for Public Health Practice

The finding that during 2021–2024 more than one in seven (15.2%) U.S. pregnant women reported drinking alcohol during the past 30 days underscores the ongoing need for comprehensive strategies to reduce alcohol consumption during pregnancy. Recommended clinical interventions include routine screening for alcohol consumption and mental health conditions, brief behavioral counseling, and referral to specialized services (*8*,*9*). Community-level approaches that include providing information about outcomes associated with alcohol consumption during pregnancy or address alcohol consumption among the general population might also help reduce prenatal alcohol exposure and prevent its associated adverse health outcomes (*4*,*5*).

## Footnotes

* Florida (2021), Kentucky (2023), Pennsylvania (2023), and Tennessee (2024) did not collect sufficient data to meet minimum requirements for inclusion in the BRFSS public use data set for those years.

† Response rates were calculated using standard definitions from the American Association for Public Opinion Research.

§ Current drinking was defined as a response of “one or more” to the question, “During the past 30 days, how many days per week or per month did you have at least one drink of any alcoholic beverage such as beer, wine, a malt beverage or liquor?” Binge drinking was defined as a response of “one or more” to the question, “Considering all types of alcoholic beverages, how many times during the past 30 days did you have four or more drinks on an occasion?”

¶ Heavy drinking (for women, eight or more alcoholic drinks in a 1-week period during the past 30 days) was calculated by BRFSS from responses to the questions, “During the past 30 days, how many days per week or per month did you have at least one drink of any alcoholic beverage such as beer, wine, a malt beverage or liquor” and, “During the past 30 days, on the days when you drank, about how many drinks did you drink on average?” Calculated Variables in the 2024 Data File of the Behavioral Risk Factor Surveillance System | CDC

** Having a usual health care provider was ascertained by a response to the question, “Do you have one person you think of as your personal doctor or health care provider?” Participants who answered “no” were asked, “Is there more than one, or is there no person who you think of as your personal doctor or health care provider?” Responses were dichotomized into one or more (yes) or none (no).

†† Frequent mental distress was defined as a response of ≥14 days to the question, “Now thinking about your mental health, which includes stress, depression, and problems with emotions, for how many days during the past 30 days was your mental health not good?” Measuring Healthy Days | CDC

§§ Among respondents with complete information on current drinking, the unweighted proportion of those with missing values for sociodemographic and health characteristics ranged from 0.4% for education to 1.7% for frequent mental distress.

¶¶ 45 C.F.R. part 46, 21 C.F.R. part 56; 42 U.S.C. Sect. 241(d); 5 U.S.C. Sect. 552a; 44 U.S.C. Sect. 3501 et seq.
